# Remote Parenting in Families Experiencing, or at Risk of, Homelessness: A Study Based on Grounded Theory

**DOI:** 10.3390/ijerph21091184

**Published:** 2024-09-05

**Authors:** Filipa Maria Reinhardt Andrade, Ana Resende, Maria Clara Roquette-Viana, Amélia Simões Figueiredo

**Affiliations:** Faculty of Health Sciences and Nursing, Center for Interdisciplinary Research in Health (CIIS), Universidade Católica Portuguesa, 1649-023 Lisbon, Portugal; anaresende@ucp.pt (A.R.); roquetteviana@ucp.pt (M.C.R.-V.); simoesfigueiredo@ucp.pt (A.S.F.)

**Keywords:** family, parenthood, parenting, vulnerable population, homeless

## Abstract

The situation/risk of family homelessness presents multiple interrelated issues. It has considerable negative consequences, namely the deterioration of the family members’ health and well-being, and alterations in the family’s dynamics, with parents sometimes being separated from their children. The aim of this research was to understand how parenting takes place in families experiencing, or at risk of, homelessness. The conducted study falls within the qualitative paradigm, using Strauss and Corbin’s version of the Grounded Theory methodology. Three main categories emerged, supported by all the participating families: “Meaning of Parenthood”, “Key Events”, and “Transition Circumstances”. These categories were translated into facilitating/inhibiting factors, within the following dimensions: “Individual”, “Family”, and “Society”. We were able to conclude that, in the population under study, parenting is restricted, being mostly exerted in a remote manner. Furthermore, it takes on different forms, depending on the specific homelessness situation/risk. In families at risk of homelessness, we identified “Remote Parenting with Maintained Parental Authority”, as well as “Restricted Parenting”, when the children still lived with their parents. On the other hand, in families experiencing homelessness, we identified “Remote Parenting with Maintained Parental Authority”, “Unilateral Remote Parenting”, “Interrupted Parenting”, and the “Total Disruption of Parenting”.

## 1. Introduction

Parenthood is an intricate concept which requires an integrative perspective from various fields of knowledge to be better understood. Being a complex transition that implies a greater vulnerability and a superior risk in terms of health, it is considered a focus point of nursing care [1,2]. It is a developmental transition in the exercise of the parental role and involves several situational transitions [1]. Nurses play a key role in preparing the person for the transition, as well as accompanying them throughout the transition process [2]. In their caring role, nurses are aware of the various types of transitions and the properties, constraints, needs, and changes that transitions entail, and prepare the client/family to better deal with these transitions by learning and acquiring new skills [1,2]. Parenting entails a set of procedures promoted by parental figures, encompassing family and community resources. Its purpose is to encourage the children’s development [3,4,5] and the internalization of expectations regarding appropriate/inappropriate parental behaviors [3]. Parenting practices are crucial in promoting the child’s health and well-being, and can contribute to the child’s development or, on the other hand, inhibit it. Homeless families face multiple and complex problems with far-reaching consequences, such as the deterioration of their members’ health, disruption of family dynamics, and the separation of parents and children [6,7]. With respect to child development, homeless families present multiple risk factors [7,8,9,10], which explains their increased vulnerability and their significance for the nursing practice. Children from homeless families are more likely to experience child abuse [11,12]. They also have a greater probability of experiencing socio-emotional, behavioral, and academic problems [9,10,13]. Moreover, high levels of anxiety and behavioral changes have been reported in these children, with parents showing difficulty in meeting their emotional and developmental needs [14]. Homeless families face numerous challenges, including the lack of play-facilitating spaces, school mobility/dropout, and behavioral/mental health problems [15]. In this sense, having parents with mental health problems, or over-reactive parents, affects the children’s neurological development that becomes more challenging. On the other hand, extremely demanding children can contribute to the emergence of mental health problems in the parents [16].

A systematic literature review carried out by Andrade et al. [17]—whose objective was to identify the parenting experiences of homeless families living in transitional shelters—found that homeless parents face complex parental experiences, influenced by insecurity, the absence of privacy, isolation, stigma, and disempowerment. Nevertheless, for some families, homelessness seems to enhance parenting by being a setting that facilitates the parental role, since it promotes family cohesion, the relationships with family members and other people, and the parents’/children’s “self”. The aforesaid review also revealed a lack of evidence on parenting in different homelessness contexts, which hinders the adjustment of nursing interventions to the families’ specific circumstances.

In Portugal, a homeless individual is someone roofless (living in a public space, a precarious location, or an emergency shelter), or houseless (living in temporary housing specifically created for that purpose) [18]. On the other hand, an individual at risk of homelessness is someone living in any of the following: unconventional and inadequate housing (e.g., caravan, precarious structure); conventional housing, temporarily, with family/friends; or insecure housing (e.g., following an eviction notice) [19]. Families who have joined the “Housing First” project are considered houseless, because the allocated houses are paid for entirely by social services. The “Housing First” project is an intervention model aimed at chronically homeless individuals, with mental health problems and/or substance abuse problems. Its objective is to integrate the individuals into the community, by granting them access to a permanent and stable (non-transitory) home [20,21,22]. This model also seeks to support the deinstitutionalization of young people living in shelters/residential care, families who survived domestic violence, psychiatric institutions, prisons, and individuals at risk of homelessness [23]. According to the report on the “National Strategy for the Integration of Homeless People” (“Estratégia Nacional para a Integração da Pessoa em situação de Sem-Abrigo”), individuals living in insecure housing (e.g., following an eviction notice) and individuals living with family and/or friends as a fallback option are considered housing risk situations, being also referred to as “hidden homelessness”. In Portugal, this is a recent phenomenon, which does not yet have an intervention as structured and consistent as explicit homelessness [19]. Hidden homelessness is also a reality in other countries. However, it never appears in official statistics and the actual living conditions of these individuals are never identified [24,25]. Family homelessness is experienced mainly by women raising their children [14,25,26]. When they cannot afford housing, such individuals are more likely to turn to family/friends. Despite not being strictly homeless, since they have a roof over their heads, these families have no legal right to the house they live in, lose their privacy, have no control over the space they inhabit, and may face overcrowding. Consequently, in some countries, they are classified as houseless [26].

The objective of the present study was to understand how parenting takes place in families experiencing, or at risk of, homelessness, in Portugal. It aimed at gaining a better comprehension regarding the parents’ life experiences, contributing to the existing scientific knowledge in the field of nursing, and aiding the development of effective health and social policies, to meet the needs of this vulnerable population.

## 2. Materials and Methods

This study is qualitative in nature, seeking to understand how parenting took place in the studied families. To better comprehend the phenomenon, we opted for the qualitative paradigm, using Strauss and Corbin’s version of the Grounded Theory methodology [27].

The research was conducted in six institutions, within the Lisbon metropolitan area, which were partners of Lisbon’s Homeless Planning and Intervention Center (“Núcleo de Planeamento e Intervenção Sem-Abrigo”—NPISA). These institutions were important resources for families experiencing, or at risk of, homelessness. They included a public bathhouse, a canteen, a social emergency reception center, and three community intervention associations—one of which was involved in the “Housing First” project. Contact with the participants was achieved through the families’ direct support teams.

The participants were not predetermined—they stemmed from the multiplicity of individuals experiencing/at risk of homelessness, which promotes theoretical consistency following discourse analysis. The final sample consisted of 30 families. In most cases, we interviewed the mother. However, in three families, we interviewed the father and, in one family, both parents were interviewed. This distribution is shown in Table 1.

We created a script for the interviews, employing open questions, to allow the parents to express themselves freely, as recommended by Strauss and Corbin [27]. The script was structured in two parts: the first part aimed to characterize the interviewee in terms of family structure and typology of homelessness situation or risk; the second part aimed to understand the process of parenting in families in a situation or risk of homelessness, through data that referred to the meaning of being a mother/father, the critical events that were highlighted during this process, and the factors that facilitate and inhibit parenting. The interviews, conducted in Portuguese, were transcribed and analyzed using the “NVivo 7” software [28]. Systematic procedures were used to process the data, such as coding (open, axial, and selective), which made it possible to discover the categories and their properties, and their relationship with the subcategories and with the central category, and ultimately arriving at the theoretical explanation of the phenomenon under study.

Ethical considerations [29,30,31] were present throughout this study, as well as compliance with national and international legislation regarding the protection of the individual’s rights [32,33,34,35]. The research began in October 2020, after due authorization from the university and its ethics committee (ref. 7392/CES/2020). The researchers ensured that the participants had adequate clarification/information about the research. After clarifying the phases of the research, the participants were asked for their written informed consent. Informed consent was obtained from all the subjects involved in the study.

To ensure the study’s reliability and validity, we employed constant comparative analysis and reflexivity, that led to the ordering of data, conceptualization, and grouping of concepts into categories. Furthermore, through peer review and external auditing [36,37,38], we were able to validate the data analysis and interpretation.

## 3. Results

The results presented in Table 1 were obtained by means of 30 interviews. They comprise information on the participants’ characterization, identifying the interviewed subject(s), the type of family structure, and the type of homelessness situation/risk. It should be noted that the families who joined the “Housing First” project were differentiated from the other houseless families, given the uniqueness of their circumstances.

**Table 1:** Characterization of the participants according to the interviewed subject(s), the type of family structure [39], and the type of homelessness situation/risk [18,19].

Interviewed Subject(s)	Type of Family Structure	Type of Homelessness Situation	Type of Homelessness Risk
E1F-M	Commune		Living with Family
E5F-M	Commune		Living with Family
E14F-M	Commune		Living with Family
E15F-M	Commune		Living with Family
E16F-M	Commune		Living with Family
E17F-M	Commune		Living with Family
E20F-M	Commune		Living with Family
E25F-M	Commune		Living with Family
E3N-P and E3N-M	Commune		Inadequate Housing
E4I-M	Commune		Insecure Housing
E8I-M	Female-led single-parent household		Insecure Housing
E19I-M	Female-led single-parent household		Insecure Housing
E2N-M	Female-led single-parent household		Inadequate Housing
E9SC-M	Female-led single-parent household	Houseless	
E1OSC-M	Female-led single-parent household	Houseless	
E235C-M	Female-led single-parent household	Houseless	
E24SC-M	Female-led single-parent household	Houseless	
E27SC-M	Female-led single-parent household	Houseless	
E3OSC-M	Female-led single-parent household	Houseless	
E28SC-M	Single person	Houseless	
E11HF-M	Single person	*Housing First*	
E12HF-P	Single person	*Housing First*	
E13HF-M	Single person	*Housing First*	
E29ST-M	Single person	Roofless	
E21N-P	Nuclear		Inadequate Housing
E22N-M	Nuclear		Inadequate Housing
E18I-M	Nuclear		Insecure Housing
E7I-M	Extended		Insecure Housing
E6F-M	Couple		Living with Family
E26SC-P	Male-led single-parent household	Houseless	

Caption—The following code letters were used to identify the interviewed subjects: E—Interview (“*Entrevista*”); M—Mother (“*Mãe*”); P—Father (“*Pai*”); SC—Houseless (“*Sem Casa*”); ST—Roofless (“*Sem Teto*”); HF—Housing First; N—Inadequate Housing (“*Habitação Não Adequada*”); I—Insecure Housing (“*Situação Habitacional Insegura*”); F—Living with Family (“*Residir com a Familia*”).

Table 1 shows that, regarding the type of homelessness situation/risk, 18 families are at risk of homelessness, while 12 families are actually homeless. Of the 18 families at risk of homelessness, 9 families (30%) are Living with Family, 5 families (17%) are in Insecure Housing and 4 families (13%) are in Inadequate Housing. Of the 12 families in a homeless situation, 11 families (37%) are Houseless and 1 family (3%) is Roofless.

As for family structure, 10 families (33%) belong to a commune, 9 (30%) to female-led single-parent households, 5 (17%) are single persons, 3 (10%) are nuclear families, 1 (3%) is a couple; 1 (3%) belongs to a male-led single-parent household, and 1 (3%) is an extended family. It can be seen that Roofless families and Housing First are all single-person, the majority of Houseless families are female-led single-parent households, the majority of families Living with Family are communes, and the families in Insecure Housing and Inadequate Housing do not have a prevalent family type.

With respect to the type of family structure, most participating families (18) consist of mothers who live alone with their children (“female-led single-parent household”) or share a house with family/friends (“commune”).

When we attempted to understand the parenting process in the studied families, we found three main categories: “Meaning of Parenthood”, “Key Events”, and “Transition Circumstances”.

The category “Meaning of Parenthood” was based on Meleis’ theory, which states that personal circumstances influence the transition. Such circumstances can either facilitate or inhibit the transitional process [1,40]. In this sense, three secondary concept-promoting categories emerged from the collected statements: “Generating”, “Providing”, and “Caring”. Regarding these secondary categories, we highlight “Caring”, due to the innovative nature of the respective findings, being reported as a type of care named “Remote Caring”, in families whose children live with a relative/were placed in residential care. “Remote Caring” is associated with a remote parental authority, which is maintained by the following: monitoring the child’s routines, as mentioned by E24SC—“*[…] so, this mother who is far away educates remotely, making video calls […] open your mouth, I want to see your teeth. Have you brushed your teeth? What did father say? He says to brush your teeth […] how are you feeling? (M)*”; having the power to decide on the purchase of goods, as shown by E28SC—“*[…] I’d get them clothes and I’d take the clothes* (to Residential Care), *I’d take the clothes… sometimes they’d be having lunch and I’d fill their table with things. (M)*”; and choosing who will take care of the child, as mentioned by E13HF—“*I know it was the best possible option… because there were other people who wanted to take care of my children then, but I decided that he should take care of them. (M)*”. Despite the distance, “Remote Caring” is also related to the presence of an “Affective Bond”, which can have various manifestations: verbal displays of affection during video calls, as referred to by E24SC—“*[…] praises, gives hugs, hugs, hugs the cell phone. (M)*”; visitation moments, as expressed by E28SC—“*[…] I’d go visit them, sometimes I’d have lunch with them and then I’d take them to school […] always playing, always talking, always… we are always meeting. (M)*”; the sacrifice of being away from the child to protect him/her, as shown by E11HF—“*[…] I’ve never done them harm […] my children don’t know I was living on the street… I won’t tell them, because it would cripple their lives […] I left her with family. (M)*”; and not giving up the child, as identified in E29ST—“*[…] almost every day I send her a message saying ‘I love you, sleep well dear’ […] she hardly ever answers […] Unconditional love, unconditional love […] I’ll never give up my daughter. (M)*”. Undoubtedly, such attitudes reflect feelings of love towards the child, regardless of the experienced separation.

“Key Events” are foreseeable experiences, or unexpected occurrences, that can be breaking/turning points for the families [1,2]. In the present study, the participants only identified negative events, which resulted in two secondary categories: “Insufficient Income”, as mentioned by E8I—“*[…] I’m starving with the kids […] I’m having a very bad time, a very bad time […] my son asks me: mama let’s go out, just… play a bit […] I can’t… I… I have nothing (M)*”; and “Separation from the Children”, as emphasized by *E28SC—*“*[…] the father tried to kill me […] my children were taken away from me at the time* (they went to Residential Care) *because I wasn’t psychologically prepared to be with my children. (M)*”.

The category “Transition Circumstances” is related to numerous factors that, in some way, hinder/facilitate the execution of the parental role in the studied families. This category is based on Meleis’ Transition Theory, encompassing personal, community, and social elements [40]. Accordingly, it resulted in three secondary categories: “Individual”, “Family”, and “Society”.

Within the category “Individual”, we found different dimensions: “Spirituality”, verified in E14F—”*[…] first I pray to Jehovah, I ask that I could stay calm* (to set limits on teenage daughter) *then I can (M)*”; “Adaptation Skills”, as verified in E2N—“*[…] I’ve never been to the movies with the girls, I don’t know what it’s like to go to the movies… because there’s no feeling left […] the feeling that it’s not possible today, to accept it as it is, so as not to suffer (M)*”; “Communication Skills”, referred to by E7I—“*Mother and daughter are friends and they’ve been talking, exchanging advices between mother and daughter, even though sometimes there’s a disagreement, that’s typical of life… then later on, we come to terms with it and return to the conversation. (M)*”; and “Motivation”, verified in E30SC—“*[…] I think I’m a fighting mother. I’m a warrior (M)*”, are the facilitating factors, while “Altered Health and Well-Being”, found in E7I—“*[…] when I’m in bed rest, I get sad […] because I think about the girls, I think about the mother, that I’m their support and they need me, and then I feel really devastated […] sometimes, I want to give up, on everything and everyone. (M)*”, is an inhibiting factor in parenting.

The category “Family” refers to the support, or lack of it, from the extended family, which facilitates/hinders the parental role’s effectiveness [40]. In this sense, “Parental Support” emerged as a facilitating factor, through direct childcare, emotional support, financial support, and home maintenance support, as mentioned by E1F—”*[…] if I’m not, I’m not at home and she’s there, she can take care of my children… yes look after them… yes… they stay with me, I also leave them with her or S. (M)*”. On the contrary, “Conflicts” (with or without relationship rupture), found in E12HF—“*[…] I have a family, but I don’t get along with them […] As we had no one to take care of the children, they went to the institution. (P)*”; and “Geographical Distancing”, verified in E15F—“*In Africa, we have a lot of people at home […] There, you have plenty of help. You don’t need much to raise your child […] you don’t have to pay. (M)*”, were recognized as inhibiting factors.

As to the category “Society”, three dimensions were defined—“Social Policies”, “National Health Service”, and “Education”. Concerning “Social Policies”, we emphasize the relevance of the following: housing, as referred to by E27SC—“*[…] It’s so good here. It does me so much good, that I even forget I’m in a reception center. […] she likes it here, a lot. (M)*”; financial support, as mentioned by E23SC—“*When I wasn’t working, Social Security supported me. (M)*”; and the legal framework, as verified in E22N—“*I managed to get a job, which granted me access to the documentation, and then I was able to get a studio apartment […] That was when I brought them, as soon as possible. (M)*”. With respect to “Education”, we call attention to formal/informal information channels, as well as the logistics of educational resources (e.g., the existence/inexistence of nursery/preschool vacancies, the distance between housing, nursery/preschool/school, and work, and the compatibility between working hours and nursery/preschool/school functioning hours), as we can find in E2N—“*[…] they* (the school) *scheduled* (the meeting) *for when I’m still leaving work […] I don’t even have time, like… to… go somewhere… because my days off are always Mondays and on Mondays they’re at school. (M)*”. “National Health Service” refers to the support given by the family health unit, the hospital, and the SNS 24 line, at the three levels of prevention, as identified in E14F—“*It was at the health center* (teaching the introduction of food). *When I took her for a vaccination, they showed me certain things I had to do. (M)*”.

While the “National Health Service” acted exclusively as a facilitating factor, “Social Policies” and “Education” were mostly facilitating factors, but sometimes exerted an inhibiting influence.

Further details on the three main categories can be seen in Tables S1–S3 [1,2,3,5,11,17,18,19,20,21,22,23,39,40,41,42,43,44,45,46,47,48,49,50,51,52,53,54,55,56], which are available in an electronic format.

## 4. Discussion

### 4.1. Meaning of Parenthood

The secondary category “Caring” offers an unusual view of the concept in question, in which we highlight the “Remote Caring” modality. In this type of care, we can find all the dimensions of the parental role [39] that are most valued in direct care: concern for the child’s physical care, particularly feeding and hygiene; emotional development, which includes the “Affective Bond” [41,42,43] (marked by displays of affection, responsibility, communication, and self-sacrificing love); the imposition of limits and structuring rules (by monitoring the child’s routines); moral development; leisure activities; health surveillance; and keeping track of school activities/results [4,44,45,57,58,59]. “Remote Caring” integrates parental feelings and the acknowledgment of parenthood, together with a maintained identity [60,61], even when the parents are not responsible for their children’s direct care. This type of care prevails in roofless families and in families associated with the “Housing First” project. Nonetheless, it also occurs in houseless families and, to a lesser extent, in “Living with Family”/“Inadequate Housing”/“Insecure Housing” situations.

### 4.2. Key Events

The secondary categories “Insufficient Income” and “Separation from the Children” portray negative occurrences that involve multiple transitions, being experienced with great stress and family suffering [6,7]. In all the participating families, it was “Insufficient Income” that led to the homelessness situation/risk. Additionally, it hinders the child’s development (due to a lack of space, privacy, and security), and is related to deficiencies in essential/material goods, as well as difficulties in commuting/traveling [17]. By being a generator of poverty, “Insufficient Income” often leads to a “Separation from the Children”. However, it also results in new ways of maintaining the parental control/bond, through “Remote Caring”. Parental imprisonment, mental illness, and domestic violence, which result in the children’s removal, aggravate the already existing poverty situation. This reality has a considerable emotional and psychological impact on mothers and children who are victims of domestic violence [12,62]. The “Separation from the Children” is present in all the typologies of homelessness situations/risks. Nevertheless, it is more evident in families experiencing homelessness (i.e., roofless, participating in the “Housing First” project, or houseless).

### 4.3. Transition Circumstances

Within the secondary category “Individual”, the facilitating factors are more significant than the inhibiting factors, except for roofless families and families involved in the “Housing First” project, in which the inhibiting factors stand out. Several authors mention “Altered Health and Well-Being”, with an emphasis on altered mental health, as a key inhibiting factor of parenting in families experiencing, or at risk of, homelessness [7,9,25,63]. Moreover, we would like to stress the lack of personal resources experienced by roofless families and families included in the “Housing First” project. This scarcity is more noticeable in roofless situations.

Regarding the secondary category “Family”, the extended family provides crucial help in the contexts under study [64], offering financial support, emotional support, home maintenance support, and direct childcare. This encourages the child’s development by allowing stability in care and affection [59]. We found that parents turn to their extended family when they are separated from their children but continue to be involved in the parental role’s promotion/development through “Remote Caring”. “Conflicts” with relationship rupture were reported in all types of homelessness situations/risks, being more frequent in roofless families and families participating in the “Housing First” project, followed by houseless families. Such disagreements seem to be associated with domestic violence, altered mental health [65], and the use of psychoactive substances [66]. On the other hand, “Conflicts”, when occurring “without relationship rupture”, were mostly mentioned in “Living with Family” contexts, deriving from the negative aspects of sharing space. The “Geographical Distancing” that occurs in migration processes leads to a direct implementation of the parental role without family support, being reported in houseless families, as well as in “Living with Family” and “Insecure Housing” situations.

As to the secondary category “Society”, except for roofless families, the three dimensions (“Social Policies”, “National Health Service”, and “Education”) were mentioned in all types of homelessness situations/risks. The single facilitating factor reported by roofless families was “Social Policies”. In this sense, they recognized the positive influence of financial support and of the aid provided by the Commission for the Protection of Children and Young People (“Comissão de Proteção de Crianças e Jovens”—CPCJ). Nonetheless, in such families, the inhibiting factors prevailed, due to the parents’ disempowerment and their fear of losing the children [3]. This fear was also expressed by houseless families and families living in “Insecure Housing” situations. Within “Social Policies”, housing emerged as a facilitating factor for houseless families and families involved in the “Housing First” project, by allowing such families to not be roofless; to have essential goods (food, hygiene, and clothing); to have social support; and to have support in exercising the parental role while in temporary housing [7]. In some of the families participating in the “Housing First” project, it also allowed rehabilitation, which encompassed the recuperation of contact with the children [67]. On the other hand, disempowerment, due to the imposition of rules while in temporary housing, with a consequent change in the parental role’s implementation, was pointed out as an inhibiting factor [7,17,64].

### 4.4. Explanatory Summary of the Parenting Phenomenon in Families Experiencing, or at Risk of, Homelessness

**Figure 1:**
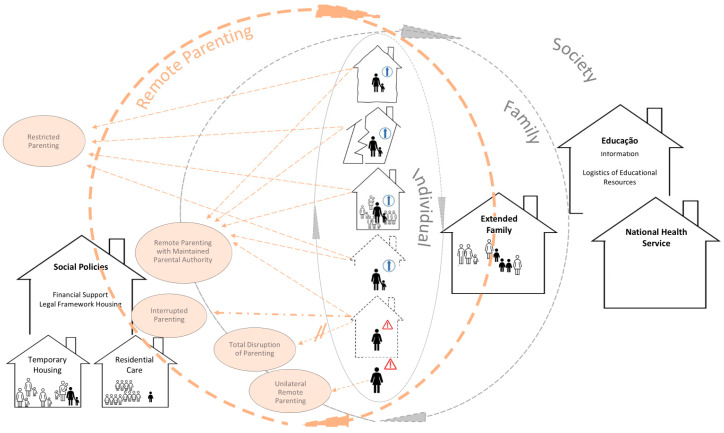
Parenting response patterns of families experiencing, or at risk of, homelessness. **Caption**—
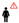
—**Roofless;** 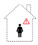
—**Housing First;** 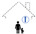
—**Houseless;** 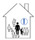
—**Living with Family;** 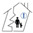
—Inadequate Housing; 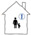
—Insecure Housing.

Families experiencing, or at risk of, homelessness exhibit various response patterns, which result in diverse forms of parenting, as can be seen in Figure 1. Most of these forms are considered “Remote Parenting”. In Figure 1, the modalities of “Remote Parenting” are represented within the respective circle, being linked to the different types of homelessness situations/risks. The circle symbolizes the process’ dynamics, crossing the main resources that support “Remote Parenting”—“Extended Family” and “Social Policies”.

In roofless families, parenting is exercised remotely, via the “Affective Bond” and the choice of who will take care of the child. The “Affective Bond” manifests itself through visitation moments and an enduring love (even when it is not reciprocated by the child—“Unilateral Remote Parenting”).

In houseless families, families participating in the “Housing First” project, and families at risk of homelessness, “Remote Parenting” is achieved through the remote presence of the parental authority, sustained by the “Affective Bond” and external resources (e.g., family)—“Remote Parenting with Maintained Parental Authority”. Regarding the families included in the “Housing First” project, there is a temporary rupture of parenting in periods of greater parental emotional disorganization (“Interrupted Parenting”), and a definitive rupture of parenting when the parents are imprisoned (“Total Disruption of Parenting”). Furthermore, such families show self-sacrificing love, by being away from the children to protect them.

In houseless families and families at risk of homelessness, when parenting is not implemented remotely, it is sustained by “Social Policies” (such as temporary housing, financial support, and a legal framework), resulting in incomplete parenthood—“Restricted Parenting”.

Hence, in families experiencing, or at risk of, homelessness, parenting is not exercised fully, but only in the possible dimensions. It is marked by unilateral relationships in roofless families, unilateral/bilateral restricted relationships in families involved in the “Housing First” project, and bilateral restricted relationships in houseless families and families at risk of homelessness. These types of parenthood have unique characteristics that should be considered when establishing educational and health policies.

The explanatory theory described above can be a starting point for further studies addressing parenthood in vulnerable families. Concerning future research, we suggest the development of studies involving parents who have experienced such situations, seeking to understand, in retrospect, how they lived and viewed parenting, as well as their coping strategies. This line of research will allow broadening the available scientific knowledge in the field of nursing.

## 5. Conclusions

Families experiencing, or at risk of, homelessness find themselves in a unique context, marked by a sequence of life events that were decisive for the parenting process (e.g., “Insufficient Income”, “Separation from the Children”). Such occurrences proved to be adverse, since the parents were unable to meet their children’s needs.

These significant life events, which did not always grant the necessary space for direct parenting, led to the discovery of new forms of parenthood, namely “Remote Caring”—a modality that allows parents to maintain their parental identity, even when the children are not under their direct care. The effectiveness of “Remote Caring” increases when it is supported by external and internal resources.

Facing a separation from their children, the parents take on the concept of parenting in a different manner with regard to the operationalization of exercising the parental role. The parental authority remains present, reflecting itself in the choice of who will take care of the child, in the monitoring of the child’s routines, and in the purchase of goods. The “Affective Bond” is also present, in the verbal displays of affection during video calls, in the visitation moments, in the self-sacrificing love of being away from the child to protect him/her, and in not giving up the child.

We concluded that parenthood exists in these families, despite not meeting all the customary requirements of the concept. In the contexts under study, parenting is considered a process, due to the instability of housing, and is sustained by personal, family, and social resources. Consequently, it is not exercised in all its dimensions, happening remotely and taking on different forms depending on the specific type of homelessness situation/risk. By exhibiting modalities of parenting that do not address all the dimensions of parenthood, these families deserve special attention from the involved organizations and professionals. As nursing professionals are considered a fundamental element in promoting the health of the population, the research carried out aims to raise awareness among nurses of this new form of parenting, with the purpose of promoting and facilitating parental authority and the affective bond, even at a distance. It is suggested that this knowledge be disseminated in clinical practice settings, namely hospitals, schools, and community health care settings. Nurses should seek to enhance the internal and external resources of these vulnerable families to exercise their parental role, paying special attention not only to training parental skills but also to spirituality, motivation, organization, expectations, the ability to communicate, enhancing conflict resolution, and health promotion, with a special focus on mental health.

Regarding social policies, it is suggested that this knowledge be integrated into the recommendations of the “National Strategy for the Integration of Homeless People”, which should be extended to the various NPISA centers.

## Data Availability

The raw data supporting the conclusions of this article will be made available by the authors on request.

## Supplementary Materials

The following additional information can be downloaded at https://www.mdpi.com/article/10.3390/ijerph21091184/s1—Table S1 (Secondary categories, dimensions, and quotes [translated from Portuguese] of the main category “Meaning of Parenthood”); Table S2 (Secondary categories, dimensions, and quotes [translated from Portuguese] of the main category “Key Events”); Table S3 (Secondary categories, dimensions, and quotes [translated from Portuguese] of the main category “Transition Circumstances”).

## References

[1] Meleis A.I. (2018). Theoretical Nursing: Development and Progress.

[2] Meleis A.I., Sawyer L.M., Im E.O., Messias D.K.H., Schumacher K. (2000). Experiencing transitions: An emerging middle-range Theory. Adv. Nurs. Sci..

[3] ICN International Council of Nurses Browser. https://www.icn.ch/.

[4] Cruz O. (2005). Parentalidade.

[5] Barroso R.G., Machado C. (2010). Definições, dimensões e determinantes da parentalidade. Psychologica.

[6] Baptista I., Marlier E. (2019). Fighting Homelessness and Housing Exclusion in Europe: A Study of National Policies.

[7] Sylvestre J., Kerman N., Polillo A., Lee C.M., Aubry T., Czechowski K. (2018). A qualitative study of the pathways into and impacts of family homelessness. J. Fam. Issues.

[8] Nichiata L.Y.I., Bertolozzi M.R., Takahashi R.F., Fracolli L.A. (2008). The Use of the “Vulnerability” Concept in the Nursing Area. Rev. Latino Am. Enferm..

[9] Perlman S., Fantuzzo J.W. (2013). Predicting risk of placement: A population-based study of out-of-home placement, child maltreatment, and emergency housing. J. Soc. Soc. Work Res..

[10] Perlman S., Sheller S., Hudson K.M., Wilson C.L., Haskett M., Perlman S., Cowan B.A. (2014). Parenting in the face of homelessness. Supporting Families Experiencing Homelessness: Current Practices and Future Directions.

[11] World Health Organization (2020). Global Status Report on Preventing Violence against Children 2020.

[12] World Health Organization (2016). INSPIRE—Seven Strategies for Ending Violence against Children.

[13] Gewirtz A.H. (2007). Promoting children’s mental health in family supportive housing: A community–university partnership for formerly homeless children and families. J. Prim. Prev..

[14] Mayock P., Neary F. (2021). Domestic Violence & Family Homelessness Challenging Homelessness.

[15] Murran S., Brady E. (2023). How does family homelessness impact on children’s development? A critical review of the literature. Child Fam. Soc. Work..

[16] Herbers J.E., DeCandia C.J., Volk K.T., Unick G.J. (2023). Profiles and predictors of neurodevelopmental functioning among young children experiencing family homelessness. Early Child. Res. Q..

[17] Andrade F.M.R., Simões Figueiredo A., Capelas M.L., Charepe Z., Deodato S. (2020). Experiences of homeless families in parenthood: A systematic review and synthesis of qualitative evidence. Int. J. Environ. Res. Public Health.

[18] Ministério do Trabalho, Solidariedade e Segurança Social Estratégia Nacional para a Integração das Pessoas em Situação de Sem-Abrigo (ENIPSSA) 2017–2023. In Presidência do Conselho de Ministros, Resolução do Conselho de Ministros n.º 107/2017, Diário da República n.º 142/2017—Série, I. https://data.dre.pt/eli/resolconsmin/107/2017/07/25/p/dre/pt/html.

[19] Estratégia Nacional Para a Integração de Pessoas em Situação Sem Abrigo (2020). Relatório: Inquérito aos Conceitos Utilizados e aos Sistemas Locais de Informação—2017. Portugal. https://www.enipssa.pt/.

[20] Martins P.D.F. (2018). Modelo Housing First Como Percursor da Mudança Social Transformativa: Uma Perspectiva Ecológica Sobre o Impacto da Percepção da Qualidade da Casa e da Escolha na Integração Comunitária, no Recovery e na Qualidade de Vida. Ph.D. Thesis.

[21] Nelson G., Kloos B., Ornelas J. (2017). Creating transformative change in community mental health: Contributions from community psychology. APA Handbook of Community Psychology: Methods for Community Research and Action for Diverse Groups and Issues.

[22] Tsemberis S., Gulcur L., Nakae M. (2004). Housing first, consumer choice, and harm reduction for homeless individuals with a dual diagnosis. Am. J. Public Health.

[23] Ornelas J., Ornelas J., Vargas-Moniz M.J., HOME_EU Consortium Study Group (2020). The HOME_EU Project on housing first as a path to end homelessness in europe. H2020_HOME_EU: Reversing Homelessness in Europe.

[24] Samzelius T. (2023). ‘It’s like they are doing injustice’: A single-mother perspective on family homelessness in sweden. Nord. Welf. Res..

[25] Forchuk C., Russell G., Richardson J., Perreault C., Hassan H., Lucyk B., Gyamfi S. (2022). Family matters in canada: Understanding and addressing family homelessness in ontario. BMC Public Health.

[26] Pleace N. (2019). Family Homelessness in Europe. Homeless in Europe. https://www.insee.fr/fr/.

[27] Strauss A., Corbin J. (2008). Pesquisa Qualitativa: Técnicas e Procedimentos para o Desenvolvimento de Teoria Fundamentada.

[28] Richards L. (2021). Handling Qualitative Data.

[29] Streubert H.J., Carpenter D.R. (2013). Investigação Qualitativa em Enfermagem: Avançando o Imperativo Humanista.

[30] Deodato S. (2014). Decisão Ética em Enfermagem: Do Problema aos Fundamentos para o Agir.

[31] ICN (2021). The ICN Code of Ethics for Nurses.

[32] (2015). Lei n.^o^ 73/2015, de 27 de Julho, Diário da República, 1ª Série, n.º 144, Diário da República nº 144/2015, Série I. https://diariodarepublica.pt/dr/detalhe/lei/73-2015-69879383.

[33] (2005). Lei n.º 12/2005, de 26 de Janeiro, Diário da República n.º 18/2005, Série I-A. https://diariodarepublica.pt/dr/detalhe/lei/12-2005-624463.

[34] (2016). Regulamento 2016/679 do Parlamento Europeu e do Conselho de 27 de Abril de 2016, Jornal Oficial da União Europeia 32. https://eur-lex.europa.eu/legal-content/PT/TXT/PDF/?uri=CELEX:32016R0679.

[35] (2015). Comissão Nacional de Proteção de Dados. Deliberação n.^o^ 1704/2015. https://www.cnpd.pt/media/grhpa2y4/del_1704_2015_investclinica.pdf.

[36] Denzin N., Lincoln Y. (2018). Introduction: Entering the field of qualitative research. Handbook of Qualitative Research.

[37] Velloso I.S.C., Tizzoni J.S. (2020). Critérios e estratégias de qualidade e rigor na pesquisa qualitativa. Cienc. Enferm..

[38] Moreira H. (2018). Critérios e Estratégias Para Garantir o Rigor Na Pesquisa Qualitativa. Rev. Bras. Ensino Ciênc. Tecnol..

[39] Figueiredo M.H. (2012). Modelo Dinâmico de Avaliação e Intervenção Familiar. Uma Abordagem Colaborativa em Enfermagem de Família.

[40] Meleis A. (2010). Transitions Theory: Middle-Range and Situation-Specific Theories in Nursing Research and Practice.

[41] Bowlby J. (1969). Attachment and Loss.

[42] Ainsworth M.D. (1985). Attachments across the life span. Bull. N. Y. Acad. Med..

[43] Kerns K.A., Schlegelmilch A., Morgan T.A., Abraham M.M. (2005). Assessing attachment in middle childhood. Attachment in Middle Childhood.

[44] Papalia D.E., Martorell G. (2021). Desenvolvimento Humano.

[45] Wilson D., Hockenberry M.J. (2016). Wong—Enfermagem da Criança e do Adolescente.

[46] Silva A.C.A., Silva E., Nunes E.N.M., Rodrigues E., Rodrigues S., Conceição V. (2022). Socialização Na Educação Infantil. Revista Ibero-Americana de Humanidades. Ciên.Edu..

[47] Fowler J. (1981). Stages of Faith: The Psychology of Human Development and the Quest for Meaning.

[48] Graffar M. (1956). Une Méthode de Classification Sociale d’Échautillons de la Population.

[49] IOM International Organization for Migration. https://www.iom.int/.

[50] (2021). Lei n.º 57/2021, de 16 agosto, Diário da República, 1ª série, n.º 158, Diário da República n.º 158/2021, Série I. https://diariodarepublica.pt/dr/detalhe/lei/57-2021-169602019.

[51] Dogra N., Lunn B., Cooper S. (2016). Psychiatry by Ten Teachers.

[52] Diário da República Lexionário. https://diariodarepublica.pt/dr/home.

[53] Duarte E.D., Braga P.P., Guimarães B.R., da Silva J.B., Caldeira S. (2022). A Qualitative Study of the Spiritual Aspects of Parenting a Child with Down Syndrome. Healthcare.

[54] Yilmaz G. (2019). Spiritual Orientation, Meaning in Life, Life Satisfaction, and Well-Being in Mothers with Disabled Children. J. Relig. Health.

[55] (1999). Lei n.º 147/99, de 1 de setembro, Diário da República 1ª série—A, n.º 204, Diário da República nº 204/1999, Série I-A. https://diariodarepublica.pt/dr/detalhe/lei/147-1999-581619.

[56] (2022). Decreto-Lei n.º 52/2022, de 4 de agosto, Diário da República n.º 150/2022, Série I-5. https://diariodarepublica.pt/dr/detalhe/decreto-lei/52-2022-187049881.

[57] Brazelton T.B. (2020). O Grande Livro da Criança. O Desenvolvimento Emocional e Comportamental dos 0 aos 3 Anos.

[58] Direção Geral da Saúde (2015). Plano Nacional de Saúde: Revisão e Extensão a 2020. DGS. http://pns.dgs.pt/.

[59] Macana E.C., Comim F., Pluciennif G.A., Lazzari M.C., Chicaro M.F. (2015). O papel das práticas e estilos parentais no desenvolvimento da primeira infância. Fundamentos da Família Como Promotora do Desenvolvimento Infantil. Parentalidade em Foco.

[60] Meighan M., Tomey A., Alligood M. (2004). Consecucao do papel maternal. Teóricas de Enfermagem e a Sua Obra—Modelos e Teorias de Enfermagem.

[61] Mercer R.T. (1995). Becoming a Mother: Research on Maternal Identity from Rubin to the Present.

[62] Lourenço L.M., Costa D.P. (2020). Violência entre parceiros íntimos e as Iiplicações para a saúde da mulher. Gerais Rev. Interinstitucional Psicol..

[63] Anderson L., Stuttaford M., Vostanis P. (2006). A family support service for homeless children and parents: User and staff perspectives. Child Fam. Soc. Work..

[64] Holtrop K., McNeil S., McWey L.M. (2015). “It’s a struggle but I can do it. I’m doing it for me and my kids”: The psychosocial characteristics and life experiences of at-risk homeless parents in transitional housing. J. Marital. Fam. Ther..

[65] Nishio A., Horita R., Sado T., Mizutani S., Watanabe T., Uehara R., Yamamoto M. (2017). Causes of homelessness prevalence: Relationship between homelessness and disability. Psychiatry Clin. Neurosci..

[66] Fletcher J.B., Reback C.J. (2017). Mental health disorders among homeless, substance-dependent men who have sex with men. Drug Alcohol Rev..

[67] Duarte T., Almas I., Ornelas J., Vargas-Moniz M.J., HOME_EU Consortium Study Group (2020). Casas primeiro program: Ten years of housing first in Portugal. Homelessness as Unfairness.

