# Supplementary material for: Remote Parenting in Families Experiencing, or at Risk of, Homelessness: A Study Based on Grounded Theory
Source: Int J Environ Res Public Health. 2024 Sep 5;21(9):1184. doi: 10.3390/ijerph21091184 (PMC11431485; doi:10.3390/ijerph21091184)
Supplement: Supplementary file 1 [file ijerph-21-01184-s001.zip › ijerph-3161044-supplementary.pdf]

Table S1: Secondary categories, dimensions, and quotes (translated from Portuguese), of the main category "Meaning of Parenthood".

**Main Category "Meaning of Parenthood"**				
**Secondary Categories**		**Dimensions**		**Quotes**
Generating				*"Born of me, will always be mine." (M; E17F-1)*
Providing				*"Parents are parents, when they can obviously support and raise their children." (P; E3N-1)*
Caring	Parental Role	Physical care	Feeding	*"[…] mother is everything. She has to take care of them, she has to give them food, clothes, the shoes they're going to wear. It's everything." (M; E14F-2)*
			Hygiene	*"[…] being a mother is… taking care of your child, to give food, to give a bath, to give everything." (M; E15F-2)*
			Sleeping and resting	*"[…] must go to bed early to get up in the morning when they have to go to school." (M; E9SC-1)*
		Leisure activities		*"Being a mother is spending time with the girls, not just working […] you have to give, to enjoy being with your children […] I still can't be a mother." (M; E2N-2)*
		School	Participation in study and meetings	*"I've never missed any of my son's meetings, despite the difficulty… always." (M; E9SC-1)*
			Creation of space for studying	*"She has a little corner there… past the kitchen we have a small balcony and that's where she studies." (M; E4I-1)*
		Security		*"[…] the child can play, but without danger […] Before giving (a bath), you put in the cold water and then you slowly put in the hot water." (M; E15F-4)*
		Health surveillance		*"Whenever they need vaccines, both the baby and the boys, then I am there… always available." (M; E5F-4)*
		Emotional and social development	Structuring rules	*"[…] They already know that, at night, they have to organize their things and, in the morning, they just get up and wash their mouths, eat breakfast and leave together for school." (M; E20F-1)*
			Imposition of limits	*"If you misbehave, if you don't eat, if you disrespect your brother, there's no game, there's no tablet, there's nothing or you go to bed earlier than usual." (M; E3N-4)*
			Affective bond	*"[…] to give affection… making them understand things, being a friend […] Not just talking, but also listening and comprehending what they need." (M; E4I-2)*

Caption – The following code letters were used to identify the interviewed subjects: E – Interview ("*Entrevista*"); M – Mother ("*Mãe*"); P – Father ("*Pai*"); SC – Houseless ("*Sem Casa*"); ST – Roofless ("*Sem Teto*"); HF – Housing First; N – Inadequate Housing ("*Habitação Não Adequada*"); I – Insecure Housing ("*Situação Habitacional Insegura*"); F – Living with Family ("*Residir com a Família*").

Table S1: Secondary categories, dimensions, and quotes (translated from Portuguese), of the main category "Meaning of Parenthood" (cont.).

Main Category "Meaning of Parenthood"				
**Secondary Categories**	**Dimensions**			**Quotes**
Caring	Parental Role	Emotional and social development	Socialization	*"[…] when there's no school, I make a point of going out with them during those two hours […] with a friend from school, a colleague from school, or even another friend they already have, right? So they can play a bit, talk, have their little chats." (M; E9SC-2)*
		Moral development		*"[…] go and study. Tell the truth. You've done something, you have to face it. You have to own it." (M; E11HF-1)*
		Spiritual development		*"[…] my children go to church every Sunday with me. […] My daughter already prays." (M; E4I-6)*
		Autonomy development		*"[…] Also by train and bus, he has a pass. He goes on his own, he's very resourceful." (M; E27SC-2)*
		Cognitive development		*"We study, we read, we watch funny videos, things to help her. Reading… I tell her to write texts to improve her handwriting." (M; E24SC-2)*
	Remote Caring	Remote parental authority	Monitoring of routines	*"[…] so, this mother who is far away educates remotely, making video calls […] open your mouth, I want to see your teeth. Have you brushed your teeth? What did father say? He says to brush your teeth […] how are you feeling?" (M; E24SC-2)*
			Purchase of goods	*"[…] I'd get them clothes and I'd take the clothes (to Residential Care), I'd take the clothes… sometimes they'd be having lunch and I'd fill their table with things." (M; E28SC-5)*
			Choosing who will take care of the child	*"I know it was the best possible option… because there were other people who wanted to take care of my children then, but I decided that he should take care of them." (M; E13HF-3)*
		Affective bond	Visitation moments	*"[…] I'd go visit them, sometimes I'd have lunch with them and then I'd take them to school […] always playing, always talking, always… we are always meeting." (M; E28SC-3)*
			Displays of affection	*"[…] praises, gives hugs, hugs, hugs the cell phone." (M; E24SC-2)*
			Enduring love	*"[…] almost every day I send her a message saying 'I love you, sleep well dear' […] she hardly ever answers […] Unconditional love, unconditional love […] I'll never give up my daughter." (M; E29ST-2)*
			Self-sacrificing love	*"[…] I've never done them harm […] my children don't know I was living on the street… I won't tell them, because it would cripple their lives […] I left her with family." (M; E11HF-4)*

Caption – The following code letters were used to identify the interviewed subjects: E – Interview (“*Entrevista*”); M – Mother (“*Mãe*”); P – Father (“*Pai*”); SC – Houseless (“*Sem Casa*”); ST – Roofless (“*Sem Teto*”); HF – Housing First; N – Inadequate Housing (“*Habitação Não Adequada*”); I – Insecure Housing (“*Situação Habitacional Insegura*”); F – Living with Family (“*Residir com a Família*”).

**Categories/Dimensions**	**Importance and meaning**
Meaning of Parenthood	was based on Meleis’ theory, which states that personal circumstances influence the transition. Such circumstances can either facilitate, or inhibit, the transitional process [1,40].
Generating	Mother's ability to conceive, to have a child.
Providing	Capacity to support the family, to bring in financial goods [39].
Caring	It refers to the family's response to the child's needs, which are expressed in the various evaluative categories of Parental Role competence adapted from Figueiredo [39].
Physical Care	This refers to the identification of children's and young people's needs in terms of knowledge about their eating patterns, hygiene, clothing, sleeping patterns and adequate rest [44,45].
Leisure Activities	This relates to the sharing of leisure time and satisfaction with the activities developed together with the child [39].
School	This is understood as the functional reorganization of the family to the new schedules of the child who has children entering school, the creation of individualized space to study, the participation of parents in the child's study activities and activities [39].
Security	This refers to identifying the child's need for physical, psychological, emotional and social safety, including accident prevention [45].
Health Surveillance	It concerns the identification of the child's needs at different levels of prevention. The first level encompasses child health surveillance, which is centred on the DGS guidelines [58].
Emotional and social development	Associated with the affective bond, the imposition of limits, structuring rules and socialization. The affective bond is at the centre of all definitions of attachment and the construction of bonds, affective relationships throughout life. It is related to the concept of attachment theory defended by Bowlby [41] and Ainsworth [42] in which there is a significant, permanent, close and responsive emotional relationship. It is also related to the discontinuity of contact, but the permanence of the emotional bond with the older child. Over the course of the individual's life cycle, changes can occur in the organization of attachment [43]. Setting limits is associated with the ability to limit or constrain behaviour [3,44,45] and structuring rules is the creation of clear and coherent rules, which signal to the child what is expected of them [44,45]. Socialization is the way a child interprets and internalizes experiences with parents, teachers, peers and cultural institutions [44]. It is related to the child's secondary socialization, which corresponds to the broadening of the child's social network after they have internalized the language, basic social rules, morals and behaviour patterns of the group to which they belong [46].

**Categories/Dimensions**	**Importance and meaning** (Cont.)
Moral Development	It corresponds to the child's ability to establish moral judgments. It implies changes in the psychic structure, configuring evolutionary stages in which the child becomes capable of moral judgments [44,45]. It is based on Kohlberg's 1968 stages of moral development [45]
Spiritual Development	It is based on Fowler's 1981 stages of spiritual development [47]
Autonomy Development	The child's ability to progressively develop independence from the various adults to whom they are attached (walking, feeding, dressing and exploring the world) until they fully develop their autonomy [5,44,45].
Cognitive Development	It consists of age-related changes in mental activity. It is based on Piaget's (1969) theory that intelligence enables children to make adaptations to the environment that increase the likelihood of survival [45].
Remote Caring	It is related to Remote Parental Authority, which is maintained by monitoring the child's routines, with the power to decide on the purchase of goods and by choosing who will take care of the child in situations where parental responsibility is transferred to a family member. This transfer happens due to the imposed or autonomous withdrawal of the child or in the case of immigration, geographical distance between home and school or eviction with the temporary handing over of the child to a relative. Remote Caring is also related to the presence of the Affective Bond, which translates into visitation moments, displays of affection through video calls, enduring love and self-sacrificing love.

Table S2: Secondary categories, dimensions, and quotes (translated from Portuguese), of the main category "Key Events".

**Main Category "Key Events"**		
**Secondary Categories**	**Dimensions**	**Quotes**
Insufficient Income	Homelessness situation/risk	*"It's complicated. […] she can't focus… it's a living room and a bedroom. I can't even tell her to go to her room and study […] I don't know how to punish her… I can't tell her… stop watching TV, go to your room." (M; E2N-7)*
	Lack of essential goods	*"When they wake up in the morning, the one who has bread will eat it and then if there's pasta, he'll eat it and, in the afternoon, the same thing […] I'm starving with the children […] I'm having an awful time, an awful time." (M; E8I-4)*
	Lack of material resources	*"[…] sometimes I get very tired because of the weight… just imagine spending the night with the child on your chest the whole night… sometimes I have cramps in my arm all day long […] the bunk bed is single and only fits one person, but we can't afford more." (M; E14F-3)*
	Difficulty in commuting/traveling	*"I can't go by public transport. I can't, it's impossible […] they say all the time that they have, that they want to spend more time with me, but unfortunately, they know it's not possible yet […] that always ends up hurting too." (M; E30SC-2)*
	Lack of medication	*"Then the child got sick, very sick and… I took her to Amadora's hospital, and they did an exam and whatnot… and they gave me a prescription to buy medicine, and that prescription is still with me… I can't buy medicine." (M; E8I-1)*
Separation from the Children	Immigration	*"[…] L. stayed in São Tomé e Príncipe, with his father […] N.'s disease had to be treated in Portugal […] I live with my daughter's illness, I live with my son's absence, I live without money, so I'm experiencing everything that's bad." (M; E24SC-1)*
	Domestic violence	*"[…] the father tried to kill me […] he shot my youngest son, who has a bullet in his head and now has a prosthetic eye… my children were taken away from me at the time (they went to Residential Care) because I wasn't psychologically prepared to be with my children." (M; E28SC-3)*
	Mental illness	*"[…] I'm an ex-drug addict… I stopped using heroin […] there was a time when… I wasn't able to leave the house, I got really depressed, I can't explain it […] We lived off the help of some neighbors, some friends […] the children were taken away from us." (P; E12HF-1)*
	Parental imprisonment	*"[…] I was arrested along with my partner, and I wasn't going to take my children to jail." (M; E13HF-2)*

Caption – The following code letters were used to identify the interviewed subjects: E – Interview ("*Entrevista*"); M – Mother ("*Mãe*"); P – Father ("*Pai*"); SC – Houseless ("*Sem Casa*"); ST – Roofless ("*Sem Teto*"); HF – Housing First; N – Inadequate Housing ("*Habitação Não Adequada*"); I – Insecure Housing ("*Situação Habitacional Insegura*"); F – Living with Family ("*Residir com a Família*").

Categories/Dimensions	Importance and meaning
Key Events	They are unexpected events or occurrences that can be breaking points and turning points in families [2].
Insufficient Income	This refers to salaries less than or equal to the national minimum wage, pensioners or retirees, uncertain salaries, public or private assistance/benefits (such as subsidies, guaranteed minimum income). This designation is based on the Graffar Scale [48]. The consequence of Insufficient Income often leads to parents being unable to meet their child's needs and is highlighted in the literature as a factor of family stress [17].
Homelessness situation/risk	According to the report on the "National Strategy for the Integration of Homeless People" ("*Estratégia Nacional para a Integração da Pessoa em situação de Sem-Abrigo*" - ENIPSSA) a homeless individual is someone roofless (living in a public space, a precarious location, or an emergency shelter), or houseless (living in temporary housing specifically created for that purpose) [18]. An individual at risk of homelessness is someone living in unconventional and inadequate housing (e.g., caravan, precarious structure); conventional housing, temporarily, with family/friends; or insecure housing (e.g., following an eviction notice) [19]. The situation/risk of family homelessness has considerable negative consequences, including a deterioration in the health and well-being of family members, changes in family dynamics and sometimes the separation of children [17].
Lack of essential goods	Essential goods are products considered necessary for a person's daily life. These goods are essential to meet a person's basic needs for survival and well-being. In this study, we considered the lack of food, clothing and hygiene products.
Lack of material resources	It is the lack of physical and concrete means that help achieve a goal, such as a bed, cell phone or computer for the child.
Difficulty in commuting/traveling	It's the difficulty in getting from one place to another.
Lack of medication	Considers it impossible to buy medication prescribed to treat their child's illness.
Separation from the Children	Refers to families where the mother/father does not live with the children.
Immigration	Movement of persons or populations from one area of a country to another, or from one continent to another, for permanent or temporary purposes and with the intention of working or residing [49].
Domestic violence	Situations in which the woman has suffered violence from her intimate partner and/or the child has suffered abuse. Based on Decree Law 57 [50]. It is considered violence not only when the mother and/or child suffer violence directly, but also when the child is an indirect victim, due to experiencing interparental conflicts [11]. Domestic violence has an impact on the victim's health (psychologically, emotionally and physically), as well as an economic and social impact [11].

**Categories/Dimensions**	**Importance and meaning** (Cont.)
Mental illness	Mental illness is diagnosable mental health conditions [51].
Parental imprisonment	It is the imprisonment of parents in a prison while they are in the custody and responsibility of the public authorities, in compliance with sentences in security measures of deprivation of liberty [52].

Table S3: Secondary categories, dimensions, and quotes (translated from Portuguese), of the main category "Transition Circumstances".

Main Category<br>"Transition Circumstances"		
**Secondary Categories**	**Dimensions**	**Quotes**
Individual — Spirituality	Hope	*"[…] I imagine a bright future for them, that's why we're going through these difficulties. Things will get better." (M; E4N -2)*
	Meaning of life	*"But the kids are my fuel to wake up every day and keep going." (M; E5F-1)*
	Personal beliefs	*"[…] my strength comes from God. I have a lot of faith. […] God calms me down […] God is inside me the whole time." (M; E27SC-1)*<br>*"[…] not going to the doctor straight away […] it's a liquid thing, which you put on your chest […] it stays there, because it makes things better again." (M; E14F-1)*
	Spiritual practice	*"[…] I do a reading, it touches us, we meditate and then there's a dialog between mother and daughter, and then we can understand each other… mother and daughter, love and forgiveness, yes that's what has helped us a lot, it's the word of God." (M; E7I-2)*
Motivation		*"[…] I think I'm a fighting mother. I'm a warrior." (M; E30SC-1)*
Adaptation Skills	Organization	*"[…] I wake up at 8am, get the girl ready, the little one, take her to school […] I go to work […] On the way back, I pick up the little one […] At home, I make dinner […] we have dinner, watch TV […] they go to bed […] that's the routine." (M; E2N-2)*
	Appropriate expectations	*"[…] I've never been to the movies with the girls […] because there's nothing left […] the feeling that today's not going to happen, accept things as they are, so as not to suffer." (M; E2N-1)*
Communication Skills		*"Mother and daughter are friends and they've been talking, exchanging advice between mother and daughter, even though sometimes there's a disagreement, that's typical of life… then later on, we come to terms with it and return to the conversation." (M; E7I-1)*
Altered Health and Well-Being		*"[…] when I'm in bed rest, I get sad […] because I think about the girls, I think about the mother, that I'm their support and they need me, and then I feel really devastated […] sometimes, I want to give up, on everything and everyone." (M; E7I-3)*

Caption – The following code letters were used to identify the interviewed subjects: E – Interview ("*Entrevista*"); M – Mother ("*Mãe*"); P – Father ("*Pai*"); SC – Houseless ("*Sem Casa*"); ST – Roofless ("*Sem Teto*"); HF – Housing First; N – Inadequate Housing ("*Habitação Não Adequada*"); I – Insecure Housing ("*Situação Habitacional Insegura*"); F – Living with Family ("*Residir com a Família*").

Table S3: Secondary categories, dimensions, and quotes (translated from Portuguese), of the main category "Transition Circumstances" (cont.).

**Main Category "Transition Circumstances"**			
**Secondary Categories**		**Dimensions**	**Quotes**
Family	Parental Support	Direct child care	*"[…] if I'm not, I'm not at home and she's there, she can take care of my children… yes look after them… yes… they stay with me, I also leave them with her or S." (M; E1F-9)*
		Financial support	*"[…] she helped us, she lent us the values to pay the rent." (M; E4I-4)*
		Emotional support	*"[…] I'm on Face (Facebook), I talk with my family, rest my mind a bit." (M; E24SC-2)*
		Home maintenance support	*"[…] I work all night… I wake up and she's already done everything." (M; E1F-1)*
	Conflicts	Without relationship rupture	*"[…] we live among siblings, but… it doesn't even feel like living among siblings, […] we have different ways of being and that complicates things […] It's like the house is only theirs and we're just passing through or visiting." (M; E15F-3)*
		With relationship rupture	*"[…] I have a family, but I don't get along with them […] As we had no one to take care of the children, they went to the institution." (P; E12HF-1)*<br>*"[…] she wouldn't let me see the child […] I go to the nursery without the grandmother knowing it. I go there to see the kid." (M; E17F-1)*
	Geographical Distancing		*"In Africa, we have a lot of people at home […] There, you have plenty of help. You don't need much to raise your child […] you don't have to pay." (M; E15F-2)*

Caption – The following code letters were used to identify the interviewed subjects: E – Interview ("*Entrevista*"); M – Mother ("*Mãe*"); P – Father ("*Pai*"); SC – Houseless ("*Sem Casa*"); ST – Roofless ("*Sem Teto*"); HF – Housing First; N – Inadequate Housing ("*Habitação Não Adequada*"); I – Insecure Housing ("*Situação Habitacional Insegura*"); F – Living with Family ("*Residir com a Família*").

Table S3: Secondary categories, dimensions, and quotes (translated from Portuguese), of the main category "Transition Circumstances" (cont.).

Main Category "Transition Circumstances"				
**Secondary Categories**		**Dimensions**		**Quotes**
Society	Social Policies	Financial support		*"When I wasn't working, Social Security supported me." (M; E23SC-1)*
		Housing	Temporary housing	*"[…] It's so good here. It does me so much good, that I even forget I'm in a reception center. […] she likes it here, a lot." (M; E27SC-2)*<br>*"They teach us (being a mother, the parental role), even because, every now and then, they give a lecture." (M; E23SC-1)*<br>*"[…] we don't get to choose, it's up to them (clothes/food) […] the children don't have much time to spend with their friends, because we have rules here." (M; E9SC-3)*
			"Housing First" project	*"[…] because they were in an institution for a little more than two years and I've only been with them… I've only been able to be with them… for two years now, since I entered the project." (P; E12HF-5)*
		Legal framework	Possession of legal documents	*"I managed to get a job, which granted me access to the documentation, and then I was able to get a studio apartment […] That was when I brought them, as soon as possible […] they didn't have a medical appointment, and I don't have a family doctor either. Because I don't have a patient number. I don't even have the document." (M; E22N-2)*
			National strategy for the protection of children and young people	*"And I was afraid to ask for help, because a lot of people seek help, and they go after those people." (M; E11HF-1)*<br>*"At the time I thought it was something worse, that they (Commission for the Protection of Children and Young People) were coming to get her. But they ensured us it wasn't the case […] we understood the mistake, we were just in time, we changed." (M; E18I-1)*
	National Health Service			*"I do health surveillance, because not only does the girl have mouth problems, due to her speech, she also has heart problems, and she's currently being followed up a lot, not only at the local health care center, but also at the hospitals." (M; E18I-2)*

Caption – The following code letters were used to identify the interviewed subjects: E – Interview ("*Entrevista*"); M – Mother ("*Mãe*"); P – Father ("*Pai*"); SC – Houseless ("*Sem Casa*"); ST – Roofless ("*Sem Teto*"); HF – Housing First; N – Inadequate Housing ("*Habitação Não Adequada*"); I – Insecure Housing ("*Situação Habitacional Insegura*"); F – Living with Family ("*Residir com a Família*").

Table S3: Secondary categories, dimensions, and quotes (translated from Portuguese), of the main category "Transition Circumstances" (cont.).

Main Category "Transition Circumstances"				
**Secondary Categories**	**Dimensions**			**Quotes**
Society	Education	Formal information		*"[…] Because with the teachers' help, we understand, little by little, what should be given to the child […] and, after all, what we should also consume, right? Since it's good for us." (P; E3N-4)*
		Informal information		*"I've learned a lot from this, and I always search on the Internet, and I also ask people who have had children, to tell me how I can do it." (M; E15F-3)*
		Logistics of educational resources	Nursery/preschool vacancies	*"This one who's three and that one who's six years old aren't studying, because there are no vacancies […] I can't work." (M; E8I-5)*<br>*"I found a nursery for the baby, I put her there when she was four months old, and I started working." (M; E5F-1)*
			Distance between housing – nursery/preschool/school – work	*"[…] I arrive very tired. And I still do many journeys to pick her up from the nanny. Every day, I leave early in the morning, at 5am with her, […] It's very far." (M; E27SC-1)*<br>*"[…] I got a remote part-time job […] my part-time job allows working from home." (M; E19I-2)*
			Compatibility between working hours and nursery/preschool/school functioning hours	*"[…] they (the school) scheduled (the meeting) for when I'm still leaving work […] I don't even have time, like… to… go somewhere… because my days off are always Mondays and on Mondays they're at school." (M; E2N-2)*

Caption – The following code letters were used to identify the interviewed subjects: E – Interview ("*Entrevista*"); M – Mother ("*Mãe*"); P – Father ("*Pai*"); SC – Houseless ("*Sem Casa*"); ST – Roofless ("*Sem Teto*"); HF – Housing First; N – Inadequate Housing ("*Habitação Não Adequada*"); I – Insecure Housing ("*Situação Habitacional Insegura*"); F – Living with Family ("*Residir com a Família*").

Categories/Dimensions	Importance and meaning
Transition Circumstances	It is related to the factors that, in some way, hinder/facilitate the execution of the parental role in the studied families. This category is based on Meleis Transition Theory, encompassing personal, community and social elements [40]. Accordingly, it originated three secondary categories: “Individual”, “Family” and “Society”.
Individual	It is related to the individual factors that, in some way, hinder/facilitate the execution of the parental role in the studied families.
Spirituality	It was based on the dimensions of spirituality adapted from the study by Duarte [53]. Spirituality is the way individuals experience moments of crisis, search for meaning in life, experience connection with themselves and others, and express beliefs, values and traditions [54].
Hope	Every kind of situation lived by the family that globally strengthen the family and nurture hope towards the future of your child [53].
Meaning of life	The Meaning of Life refers to the reason for the parents' existence, and they say that their children are the reason for their existence, giving them a reason to fight and live [53]
Personal beliefs	They relate to fundamental aspects of life and faith and may or may not be associated with any religion [53]
Spiritual practice	These are situations mentioned by parents who expressed their spirituality through religious rites and practices, which facilitate the connection with the divine [53]
Motivation	It resides in behavior focused on the goal of looking after children, looking for a better life (looking for a job, a house, regularization of documents).
Adaptation Skills	It is related to the creation of appropriate expectations and the ability to organize.
Organization	It is the use of strategies to be able to respond (directly or indirectly) to the child's needs. It includes the division of household tasks, the establishment of routines and financial organization.
Appropriate expectations	It is related to expectations appropriate to the child's development and life circumstances.
Communication Skills	It resides in communication with children and with people who use or work in social resources. Communication is interactive behavior: giving and receiving information using verbal and non-verbal behaviors face to face or with synchronized or non-synchronized technological means [44].
Altered Health and Well-Being	It is based on the ICNP definition of health. Health - Status: dynamic process of adapting to and coping with the environment, meeting needs and achieving maximum potential for physical, mental, spiritual and social well-being, not merely the absence of disease or infirmity [44]

Categories/Dimensions	**Importance and meaning** (Cont.)
Family	It is related to the family factors that, in some way, hinder/facilitate the execution of the parental role in the studied families.
Parental Support	It refers to the support or lack of support from the extended family (especially the woman's mother and significant others), which in some way facilitates or hinders the parental role [40]. It is the support or lack of support given by the extended family (especially the woman's mother and significant others), which in some way facilitates or hinders the parental role [40]. It refers to direct childcare, financial support, emotional support and home maintenance support.
Conflicts	This refers to interpersonal conflicts between parents and the extended family that have a direct or indirect impact on parenting. These can be with or without a break in the relationship.
Geographical Distancing	It is related to the fact that the extended family lives far away, preventing direct support in the parental role.
Society	It is related to the Society factors that, in some way, hinder/facilitate the execution of the parental role in the studied families.
Social Policies	It concerns the political strategy aimed at situations of poverty or extreme poverty, which directly or indirectly interfere with parenting practices. It includes financial support, housing and legal framework
Financial support	This refers to the support given by the various associations and churches (essential goods, material goods, financial support); free healthcare, education and transport; support from the Social Security Institute (including family allowance, social insertion income and unemployment benefit); and alimony.
Housing	Includes housing alternatives to homelessness, such as temporary housing and “Housing First” project.
Temporary housing	This refers to families who find themselves homeless and who find short-term shelter in accommodation designed for this purpose. Temporary housing it seeks to respond to issues identified in people and families at risk of social emergency, namely housing needs, food support, hygiene and clothing, basic health care and relational, occupational and employment issues [18].
“Housing First” project	This refers to the parents integrated into the Housing First project, which is an intervention model aimed at chronically homeless individuals, with mental health problems and/or substance abuse problems. Its objective is to integrate the individuals into the community, by granting them access to a permanent and stable (non-transitory) home [20–22]. This model also seeks to support the deinstitutionalization of young people living in shelters/residential care, families who survived domestic violence, psychiatric institutions, prisons, and individuals at risk of homelessness [23].
Legal framework	Is associated with the possession of legal documents and national strategy for the protection of children and young people.

**Categories/Dimensions**	**Importance and meaning** (Cont.)
Possession of legal documents	Refers to foreigners who are in a regular or irregular situation in Portugal and is identified by parents as a factor that interferes with meeting their children's needs.
National strategy for the protection of children and young people	It is related to the system for the promotion and protection of children and young people [55]
National Health Service	It refers to the support provided by the family health unit, the hospital and the SNS 24 line, at the three levels of prevention [56].
Education	Corresponds to Formal information channels, Informal information channels and Logistics of educational resources.
Formal information	This refers to education that takes place in educational institutions, corresponding to compulsory basic education, as well as other resources of an educational nature, such as the church, through the transmission of ethical principles and moral values, and support institutions.
Informal information	Corresponds to any formative experience that is not formal, such as information shared by other mothers and access to information obtained through information technologies.
Logistics of educational resources	This refers to nursery/preschool vacancies; Distance between housing – nursery/preschool/school – work; and Compatibility between working hours and nursery/preschool/school functioning hours.
